# Supplementary material for: Balancing land use for conservation, agriculture, and renewable energy
Source: Nat Commun. 2026 Mar 7;17:3623. doi: 10.1038/s41467-026-69952-6 (PMC13096339; doi:10.1038/s41467-026-69952-6)
Supplement: Supplementary file 1 — Supplementary Information [file 41467_2026_69952_MOESM1_ESM.pdf]

Supplementary Information

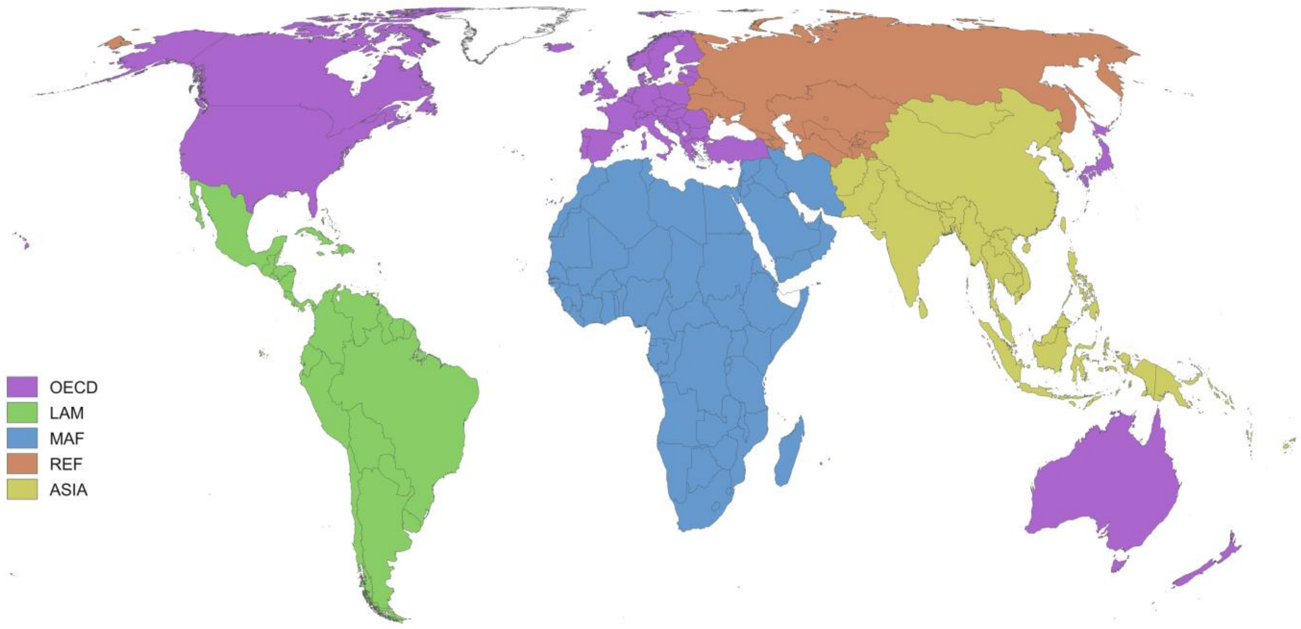

Figure S1. The five SSP Regions, as defined in the SSP database (Riahi et al., 2017) including: Asia (ASIA; yellow), Latin America (LAM; green), MAF (Middle East and Africa; blue), OECD countries (OECD; purple) and the countries from reforming economies of Eastern Europe and the former Soviet Union (REF; orange). Country boundary data are from Natural Earth (www.naturalearthdata.com).

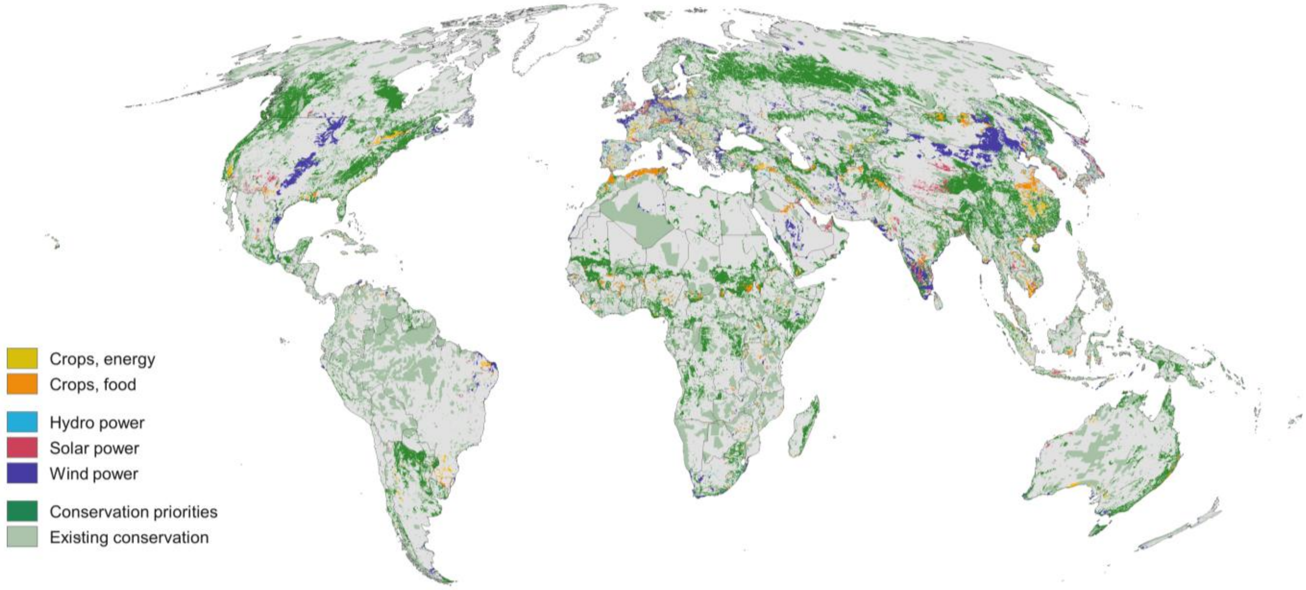

Figure S2. Land allocated for conservation and development in the Production-First planning scenario. Each country has 30% of its land allocated to conservation, including current conservation areas. Development includes food crops (orange), energy crops (yellow), solar power (pink), wind power (purple), hydro power (blue) and conservation includes existing conservation (light green) and conservation priorities (dark green). Demand is based on 2050 projections. Current conservation areas include protected areas and other effective conservation measures (UNEP-WCMC and IUCN, 2021). Country boundary data are from Natural Earth (www.naturalearthdata.com).

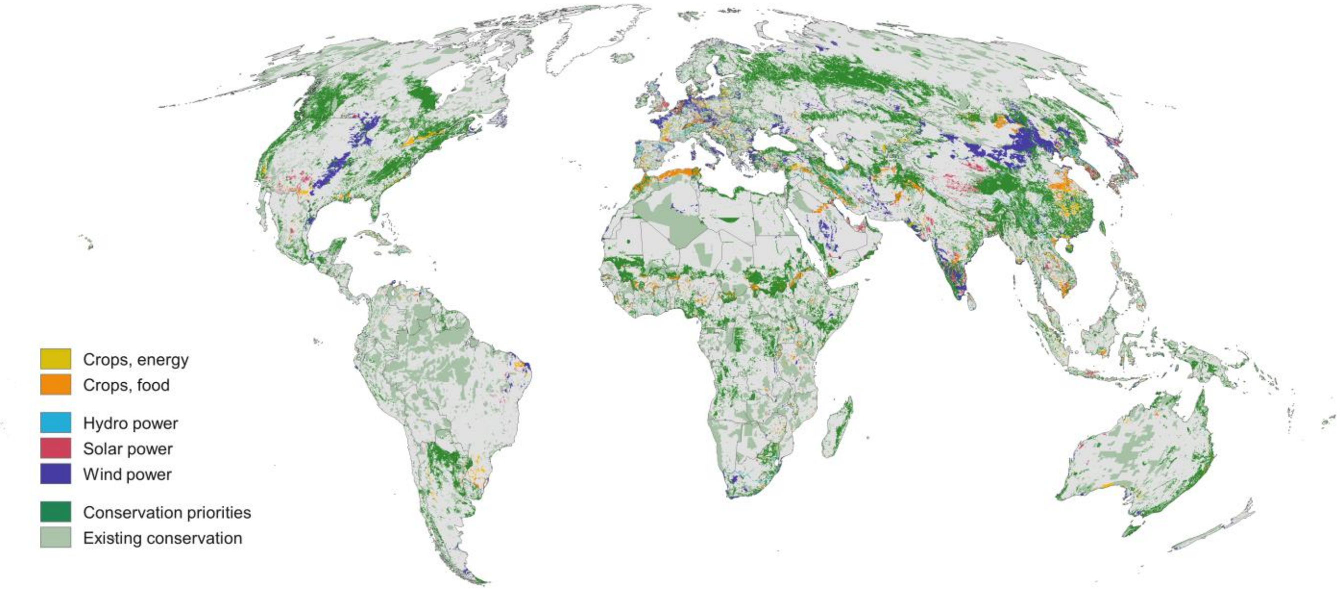

Figure S3. Land allocated for conservation and development in the Nature-First planning scenario. Each country has 30% of its land allocated to conservation, including current conservation areas. Development includes food crops (orange), energy crops (yellow), solar power (pink), wind power (purple), hydro power (blue) and conservation includes existing conservation (light green) and conservation priorities (dark green). Demand is based on 2050 projections. Current conservation areas include protected areas and other effective conservation measures (UNEP-WCMC and IUCN, 2021). Country boundary data are from Natural Earth (www.naturalearthdata.com).

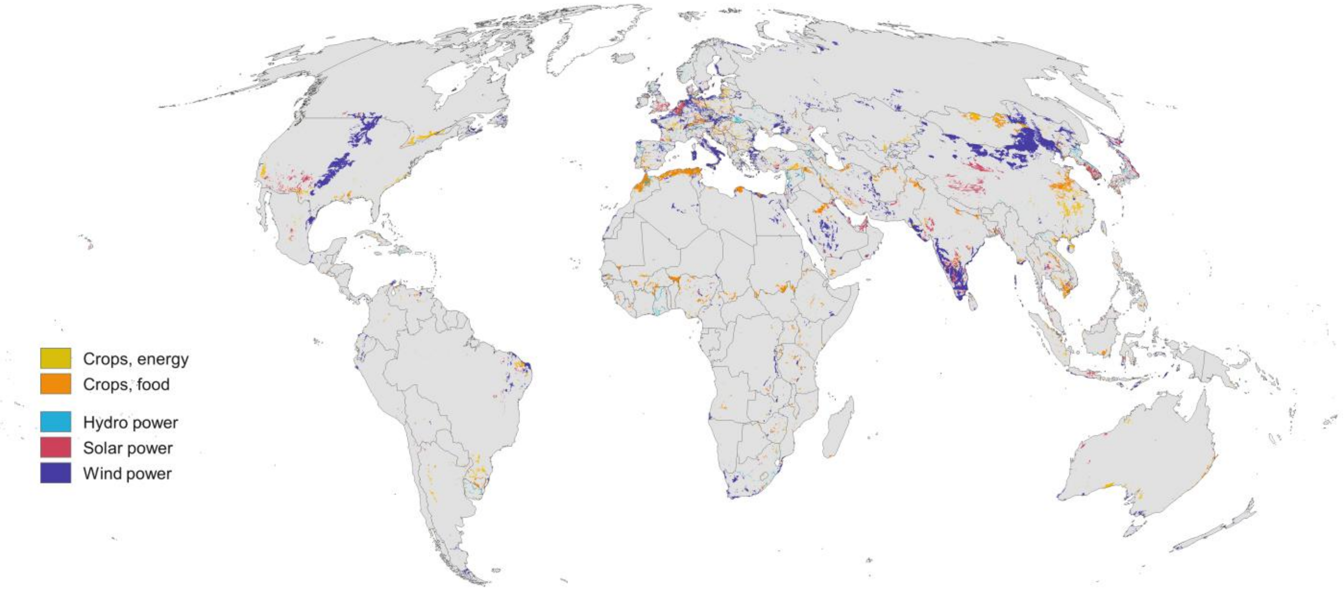

Figure S4. Land allocated for development without a constraint for only allocating 30% of land to conservation. Development sectors include food crops (orange), energy crops (yellow), solar power (pink), wind power (purple), and hydro power (blue). Country boundary data are from Natural Earth (www.naturalearthdata.com).

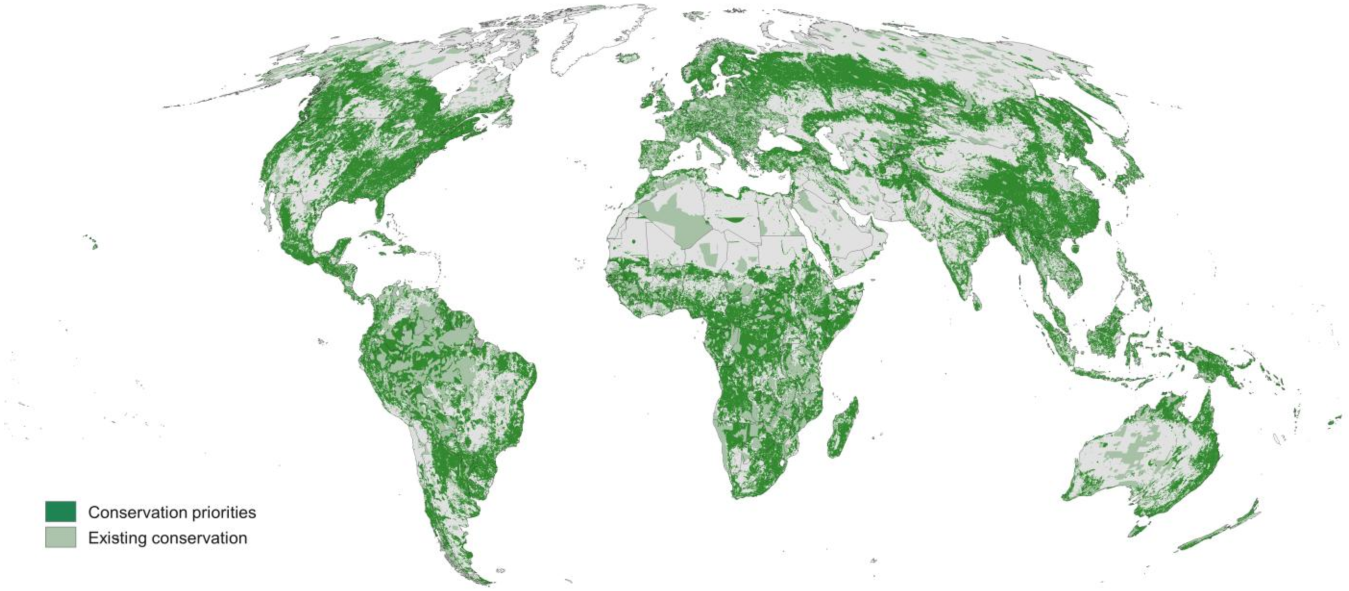

Figure S5. Conservation priorities (dark green) for meeting all country-level nature targets for biodiversity and nature's contribution to people (NCP), without a constraint for only allocating 30% of land to conservation. Current conservation areas (light green) include protected areas and other effective conservation measures (UNEP-WCMC and IUCN, 2021). Country boundary data are from Natural Earth (www.naturalearthdata.com).

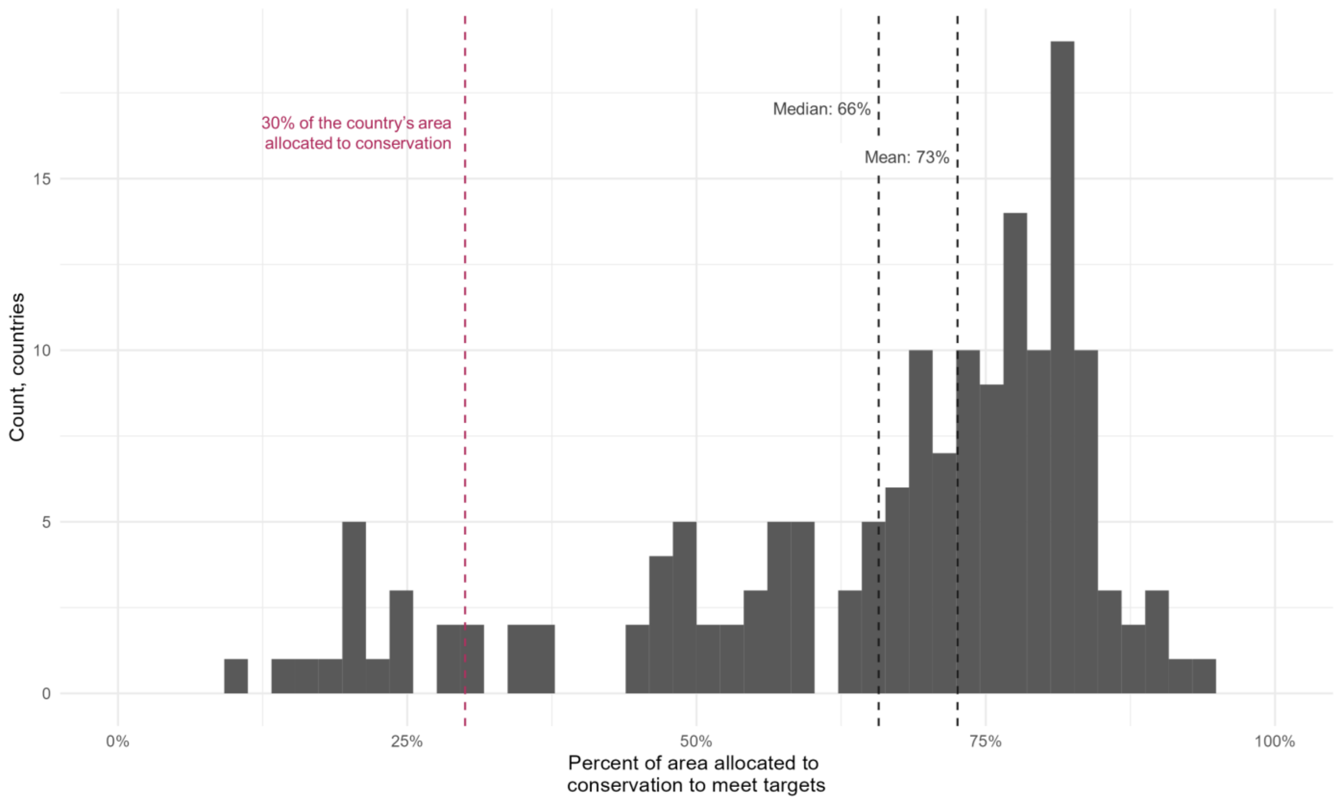

Figure S6. Percent of area allocated to conservation to meet targets in the Nature-First planning scenario. Area includes current conservation areas in addition to new land allocated. See results for all countries and scenarios in Supplementary Data 1.

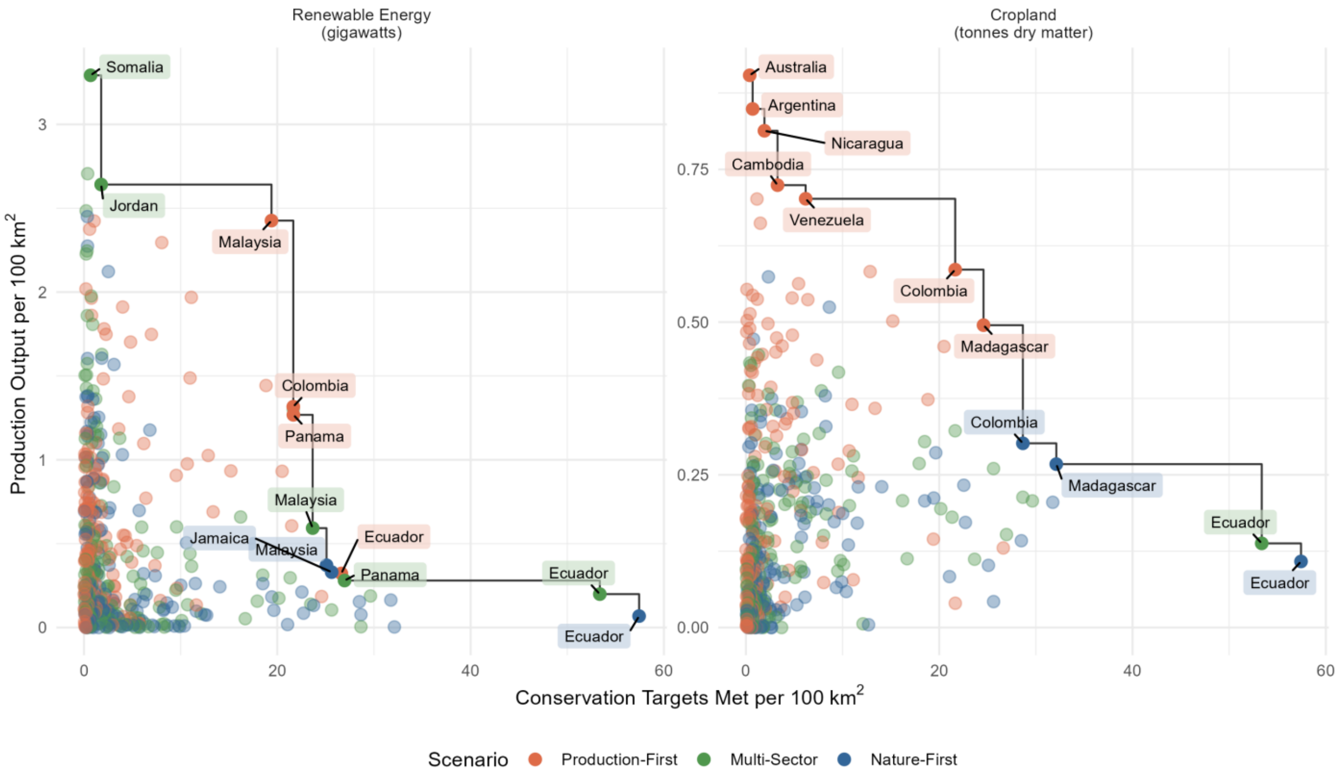

Figure S7. Pareto optimality frontier comparing production output (renewable energy in left panel, cropland in right panel) to conservation targets met per 100km^2 for all countries. Production-first is denoted in orange, multi-sector in green, and nature-first in blue. All scenarios reflect those run without a constraint on land allocated for conservation.

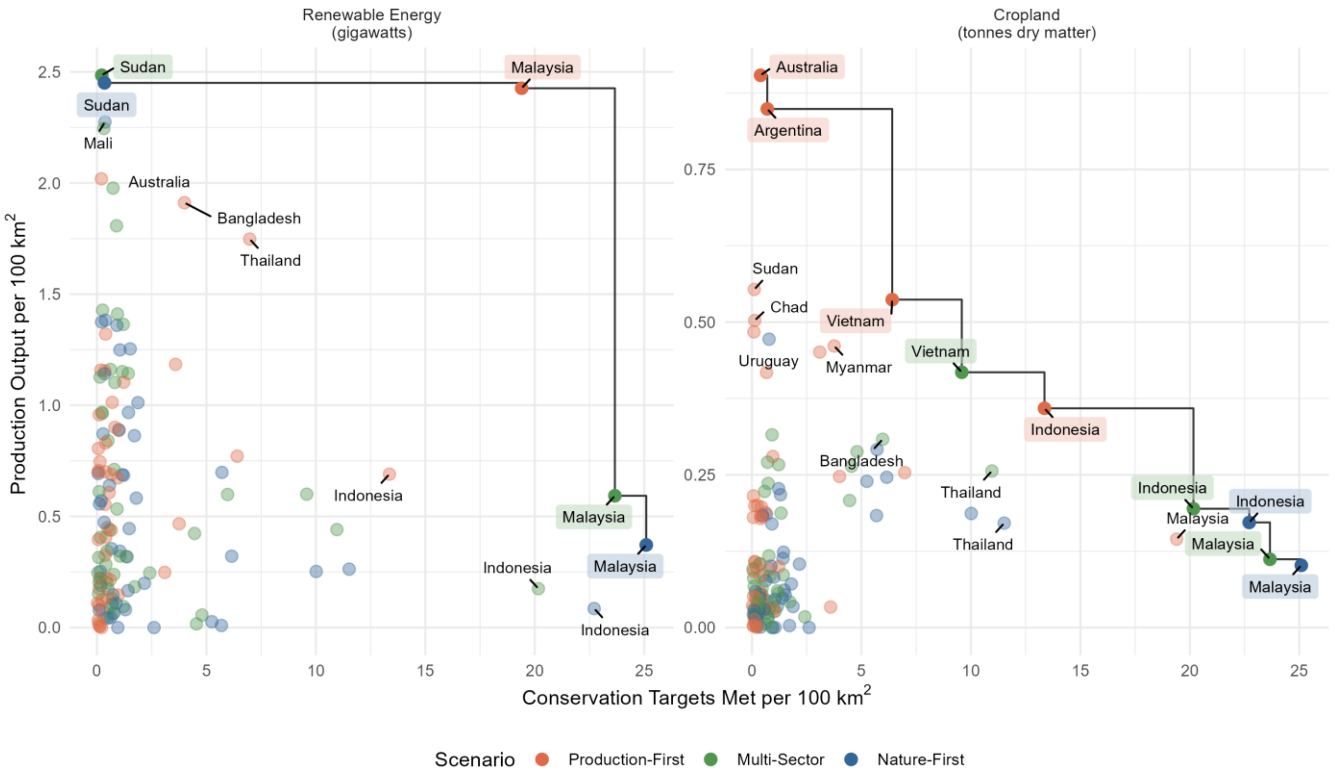

Figure S8. Pareto optimality frontier comparing production output (renewable energy in left panel, cropland in right panel) to conservation targets met per 100km^2 for countries with the highest area of overlapping priorities for conservation and production (i.e., countries identified in Fig. S10 right panel). Production-first is denoted in orange, multi-sector in green, and nature-first in blue. All scenarios reflect those run without a constraint on land allocated for conservation.

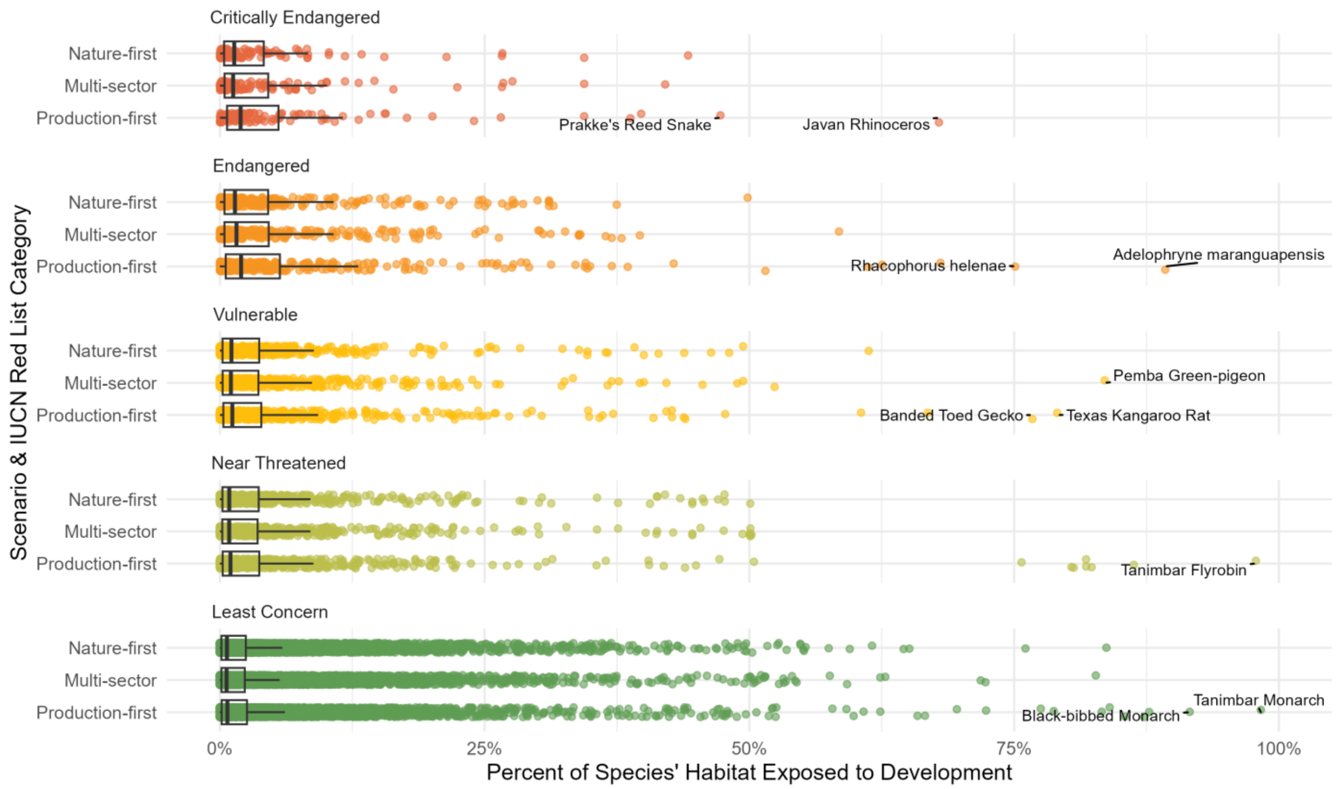

Figure S9. Percent of each species' habitat exposed to development across scenarios. In the box plots, the center lines denote median values, boxes extend from the 25th to the 75th percentile of each group's distribution, and the whiskers extend to a maximum of 1.5 times the interquartile range. Colors reflect the IUCN Red List Category of each species: Least Concern (dark green), Near Threatened (NT; light green), Vulnerable (VU; yellow), Endangered (EN; orange), and Critically Endangered (CR; red).

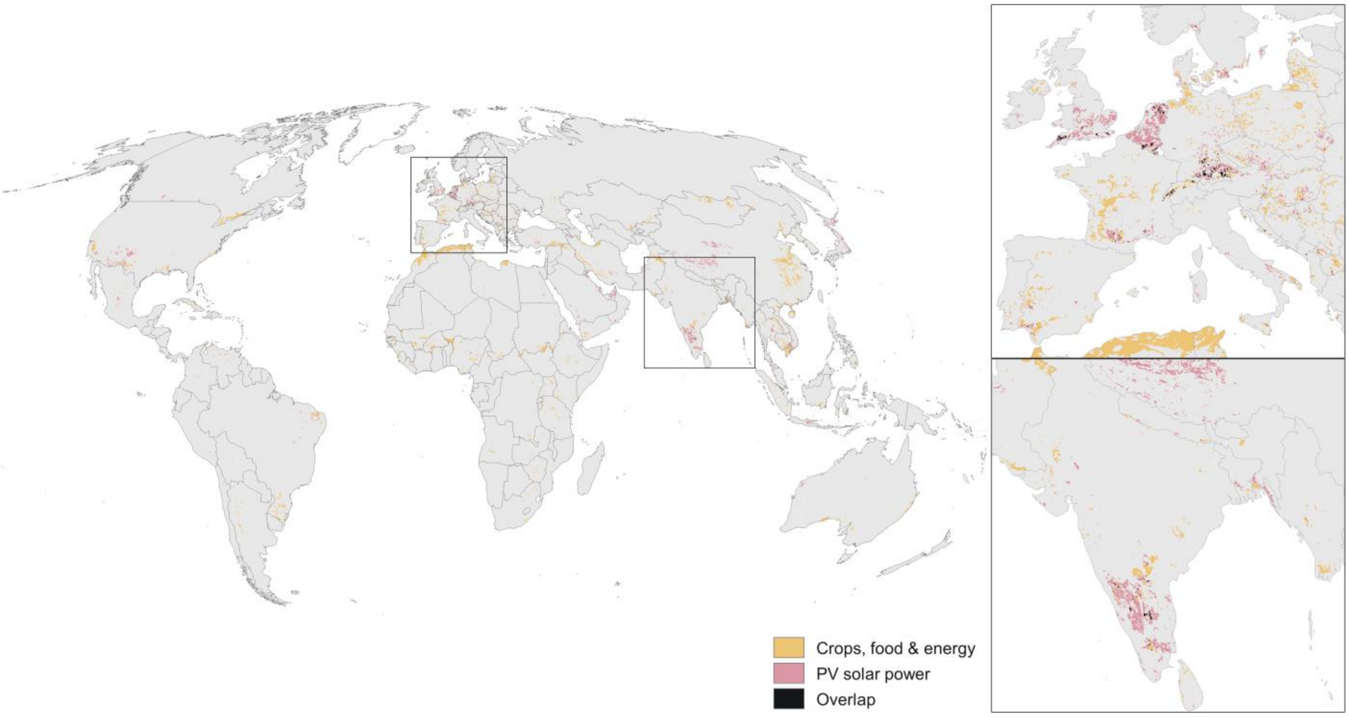

Figure S10. Global overlap (black) between land allocated for photovoltaic solar energy (pink) and for crops (orange; includes crops for food and energy) when allocated independently. Country boundary data are from Natural Earth (www.naturalearthdata.com).

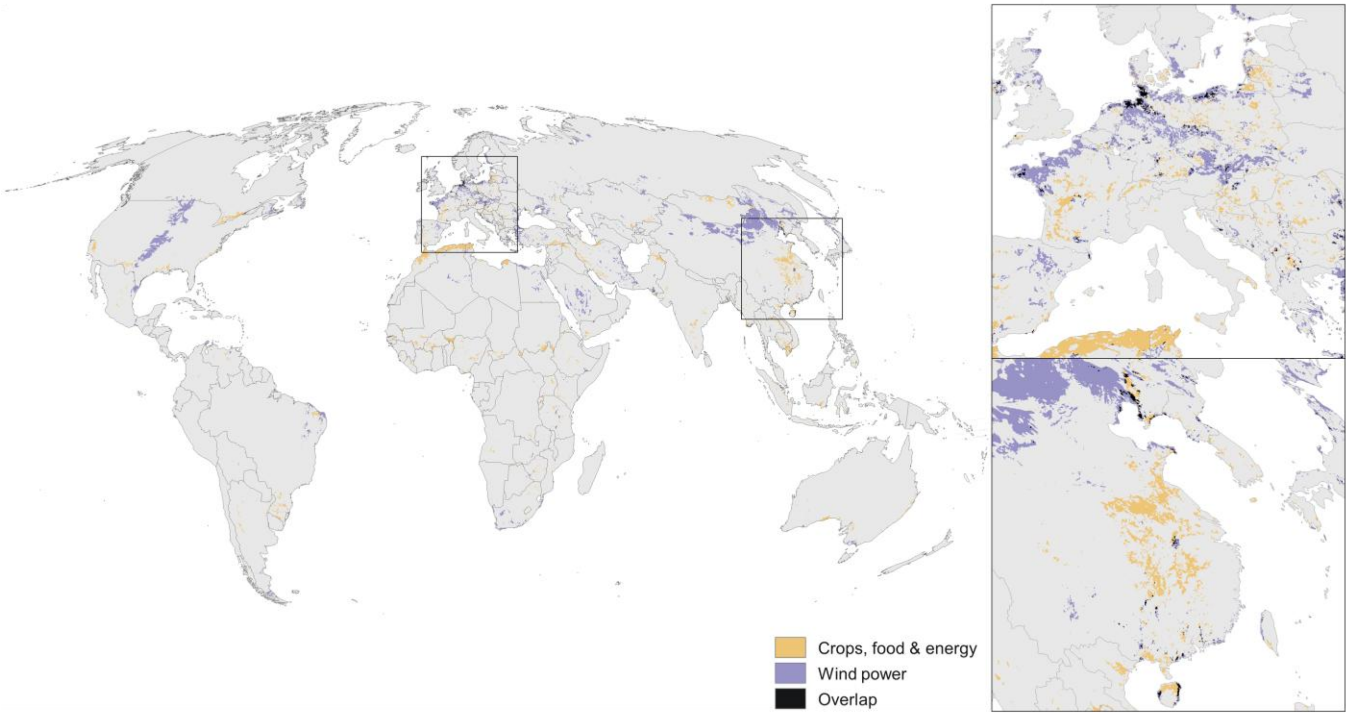

Figure S11. Global overlap (black) between land allocated for wind energy (purple) and for crops (orange; includes crops for food and energy) when allocated independently. Country boundary data are from Natural Earth (www.naturalearthdata.com).

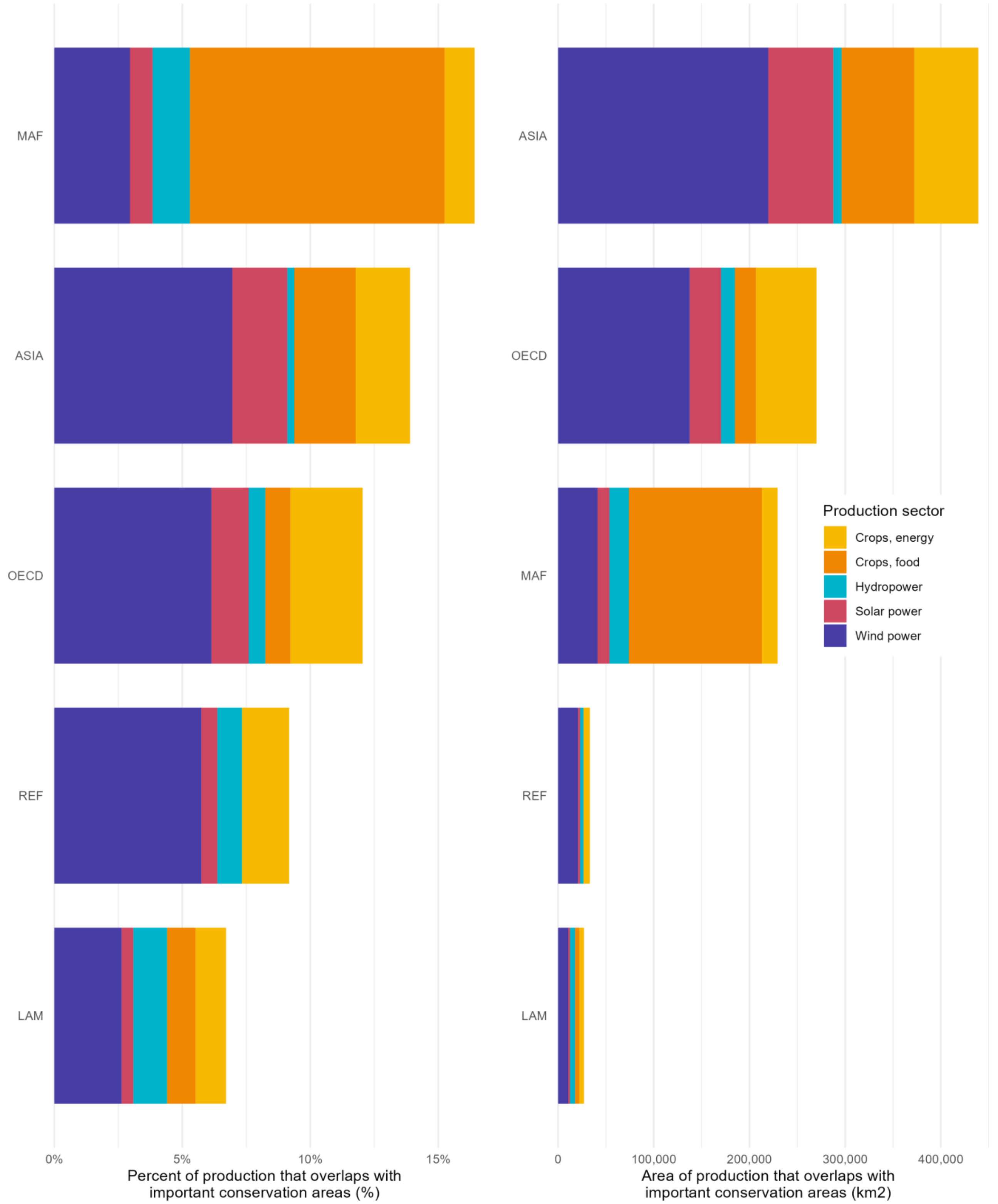

Figure S12. The left panel is percent of allocated production that overlaps with important conservation areas in the "30% conservation" runs. The right panel is the area of development that overlaps with the conservation areas. Sectors include food crops (orange), energy crops (yellow), solar power (pink), wind power (purple), and hydro power (blue).

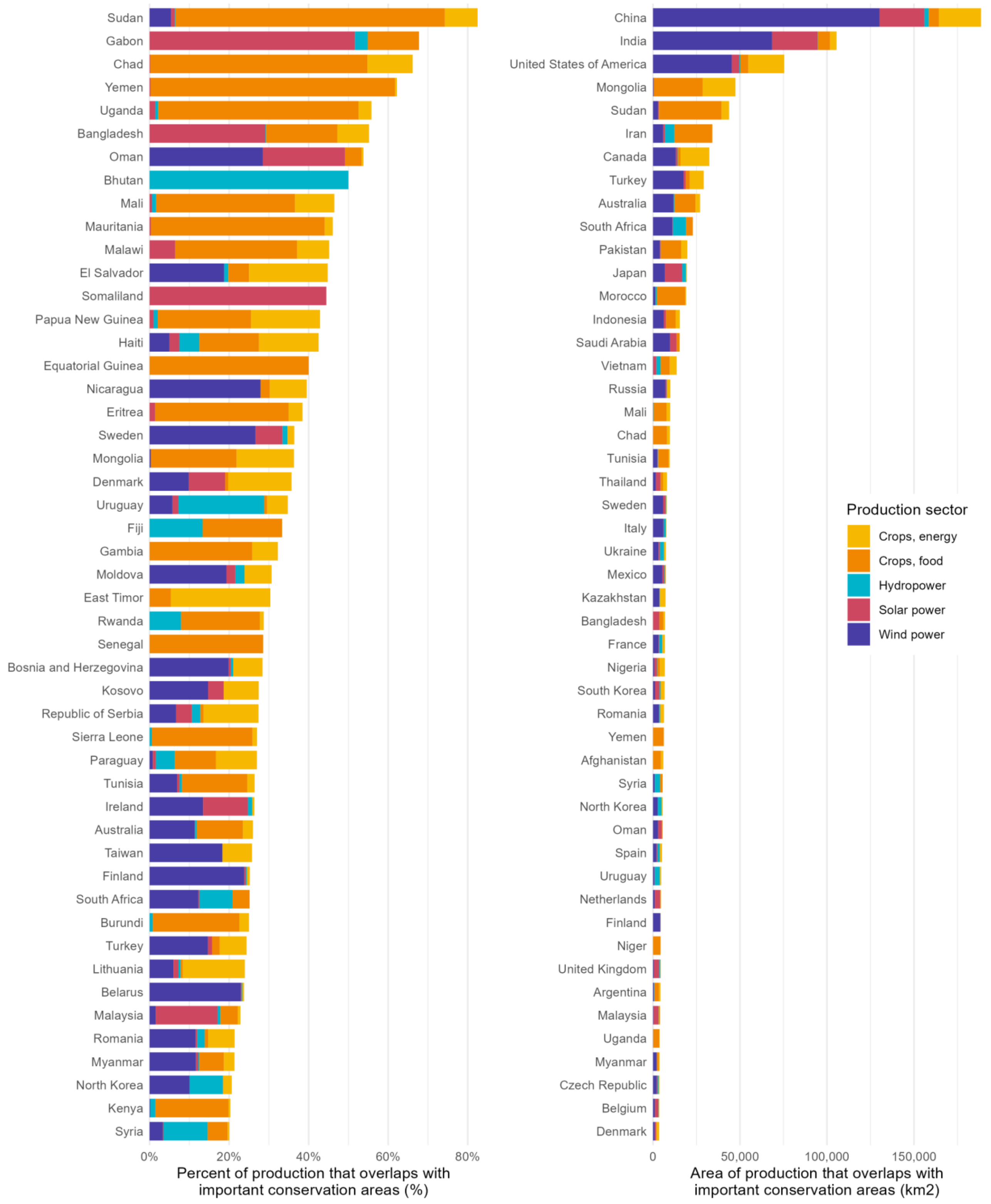

Figure S13. Left panel is percent of allocated production that overlaps with important conservation areas in the "30% conservation" runs. Right panel is the area of development that overlaps with the conservation areas. The top 50 countries for each are shown. Land allocation for all countries can be found in Supplementary Data 1. Sectors include food crops (orange), energy crops (yellow), solar power (pink), wind power (purple), and hydro power (blue).

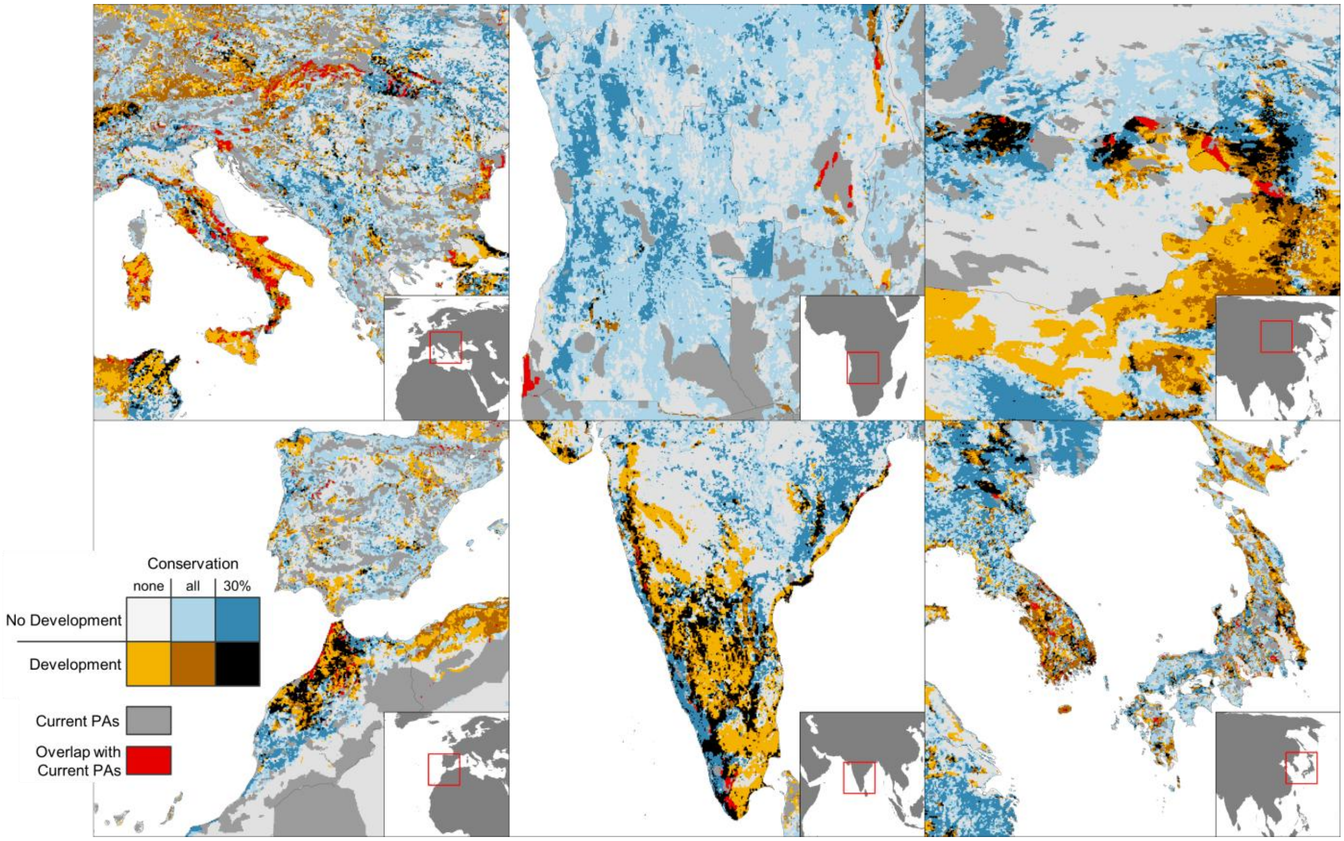

Figure S14. Area of potential conflict between additional land allocated to conservation under the Nature-First planning scenario and development under the Production-First scenario. For conservation, light blue reflects the area needed to get as close as possible to meeting all country-level nature targets. In contrast, the dark blue is the optimal area for conservation when constrained to 30% of land. Yellow reflects land that was allocated for development. Any overlap of land allocated for development and conservation is in the brown and black categories, depending on whether the development overlapped with the conservation areas needed to get as close as possible to meeting nature targets (brown) or conservation areas constrained to 30% of land (black). Overlap of development and current conservation areas (PAs) is highlighted in red. Demand is based on 2050 projections. Existing conservation areas (grey) include protected areas and other effective conservation measures (UNEP-WCMC and IUCN, 2021). Country boundary data are from Natural Earth (www.naturalearthdata.com).

Table S1. Additional land allocated for conservation and production per target met under each scenario for scenarios run with a 30% constraint on land allocated for conservation.

Scenario	Mean Additional Land Allocated for Conservation per Conservation Target Met	Mean Additional Land Allocated for Production per Production Target Met
**Production-First**	5,171.60 km^2	7,154.78 km^2
**Multi-Sector**	5,170.49 km^2	8,005.35 km^2
**Nature-First**	5,134.41 km^2	8,254.85 km^2

Table S2. Overview of data sets and their uses.

Data set	Overview & Use	Reference
General		
**Current conservation areas**	Current conservation areas were compiled from the World Database on Protected Areas and the World Database on Other Effective Area-Based Conservation Measures. In the conservation land allocation, conservation built upon existing conservation areas to meet targets. In the development land allocation, there was an additional cost associated with allocation land on current conservation areas.	UNEP-WCMC and IUCN, 2021
**Land availability**	The global human modification map (HM) was used to identify land not available for additional land allocation due to existing intensive human activities. This threshold was set at values above 80% modified following Johnson et al., 2021.	Kennedy et al., 2019
Conservation		
**Current species habitats**	Current species habitats are based on Area of Habitat (AOH) – which is derived from Red List expert range maps that have been filtered for intact habitat based on up-to-date satellite data as well as known elevation limits. See Methods for details on creation of the AOH layers. We overlap these data with the future climatic suitability data at the species level to define the biodiversity features. For species that did not have an overlap between their current AOH and their future climatic suitability, their current AOH was used.	IUCN, 2021 and Birdlife, 2020
**Future species climatic suitability**	Future climatic suitability in an SSP 3, RCP 7.0 2050 scenario. We overlap these data with the current species habitat data at the species level to define the biodiversity features. For species that did not have an overlap between their current AOH and their future climatic suitability, their current AOH was used.	Hannah, Roehrdanz, Marquet, et al., 2020
**Carbon and other nature's contributions to people**	Carbon is represented by vulnerable terrestrial carbon storage, which represents the spatially explicit, above-ground, and below-ground carbon that could be lost in a typical disturbance event. Additional NCP features included six data layers that were most relevant to area-based	Noon et al., 2021 (carbon only) and Chaplin-Kramer et al., 2020

	conservation efforts and were publicly available from Chaplin-Kramer et al., 2022: nitrogen retention for water quality regulation, sediment retention for water quality regulation, coastal risk reduction, pollinator habitat sufficiency for pollination-dependent crops, flood regulation, and access to nature. Each layer was considered an individual feature when allocating land for conservation.	
Development		
**Future demand**	The targets for each development sector were retrieved from the shared socioeconomic pathway (SSP) database, defined as future sector-specific demand for energy in gigawatts (PV solar, CSP solar, hydro, and onshore wind) and tonnes of dry matter (crops for fuel and crops for food) within the SSP1 , "Sustainability" narrative for the year 2050 (Riahi et al., 2017). These data are available at the region level. Regions include Asia (ASIA), Latin America (LAM), Middle East and Africa (MAF), Organization for Economic Co-operation and Development countries (OECD), and the countries from reforming economies of Eastern Europe and the former Soviet Union (REF).	Riahi et al., 2017
**Country-level share of future demand**	The database provides demand at the region level, which we allocated to each country based on their projected market share. For wind and solar power sectors, the projected market share was calculated from projections (Jacobson et al., 2019). Jacobson et al., 2019 does not include projections for all countries; thus, the market share for countries not considered in their analyses and projections for market share for hydropower were calculated based on current market share (IRENA, 2023). Country-level market share for cropland is calculated from change in cropland between 2050 and 2020 in an SSP 1 scenario (Chen et al., 2022). The projected market share for cropland was used for bioenergy as well, as Chen et al., 2022 do not differentiate between whether cropland is used for food or energy crops.	Jacobson et al., 2019 (wind, solar); IRENA, 2023 (hydro); and Chen et al., 2022 (cropland)
**Potential yield**	Potential yield for photovoltaic solar power, concentrated solar power, onshore wind power, hydropower, and crops for energy are from Oakleaf et al., 2019. For food crops, potential yield is calculated as the maximum potential yield under a 2050 RCP 6.0 scenario (Fisher et al., 2021) across four food crops that occupy the most land globally: maize, rice, soy, and wheat (FAOSTAT, 2016). Potential yields were used as features in allocation land for development.	Oakleaf et al., 2019 (energy) and FAO GAEZ (cropland)
**Feasibility limitations**	Feasibility limitations for siting land for development were based on the inverse of the sector-specific development potential index (DPI). The DPI is a suitability index for development potential based on spatially-explicit available resources, siting constraints (i.e., land cover, slope), and drivers for development (i.e., major roads, rails, ports, powerlines).	Oakleaf et al., 2019
