# Supplementary material for: Balancing land use for conservation, agriculture, and renewable energy
Source: Nat Commun. 2026 Mar 7;17:3623. doi: 10.1038/s41467-026-69952-6 (PMC13096339; doi:10.1038/s41467-026-69952-6)
Supplement: Supplementary file 2 — Description of Additional Supplementary Files [file 41467_2026_69952_MOESM2_ESM.pdf]

Description of Additional Supplementary Files

File Name: Supplementary Data 1
Description: Excel spreadsheet with country-level land allocation for all scenarios. Features include Country, Planning Scenario, Conservation Constraint, Sector, Area (sq km), and Area (percent).
